# The advanced lung cancer inflammation index (ALI) predicted the postoperative survival rate of patients with non-small cell lung cancer and the construction of a nomogram model

**DOI:** 10.1186/s12957-024-03432-3

**Published:** 2024-06-14

**Authors:** Shixin Ma, Zongqi Li, Lunqing Wang

**Affiliations:** 1 Graduate School, Dalian Medical University, Dalian, 116000 Liaoning China; 2 Department of Thoracic Surgery, Qingdao Municipal Hospital, No.5 Donghai Middle Road, Qingdao, 266071 Shandong China

**Keywords:** Non-small cell lung cancer, Advanced lung cancer inflammation index, Prognosis, Nomogram

## Abstract

**Objective:**

To investigate the prognostic significance of the advanced lung cancer inflammation index (ALI) in patients with operable non-small-cell lung carcinoma (NSCLC). By constructing the nomogram model, it can provide a reference for clinical work.

**Methods:**

A total of 899 patients with non-small cell lung cancer who underwent surgery in our hospital between January 2017 and June 2021 were retrospectively included. ALI was calculated by body mass index (BMI) × serum albumin/neutrophil to lymphocyte ratio (NLR). The optimal truncation value of ALI was obtained using the receiver operating characteristic (ROC) curve and divided into two groups. Survival analysis was represented by the Kaplan-Meier curve. The predictors of Overall survival (OS) were evaluated by the Cox proportional risk model using single factor and stepwise regression multifactor analysis. Based on the results of multi-factor Cox proportional risk regression analysis, a nomogram model was established using the R survival package. The bootstrap method (repeated sampling 1 000 times) was used for internal verification of the nomogram model. The concordance index (C-index) was used to represent the prediction performance of the nomogram model, and the calibration graph method was used to visually represent its prediction conformity. The application value of the model was evaluated by decision curve analysis (DCA).

**Results:**

The optimal cut-off value of ALI was 70.06, and the low ALI group (ALI < 70.06) showed a poor survival prognosis. In multivariate analyses, tumor location, pathological stage, neuroaggression, and ALI were independently associated with operable NSCLC-specific survival. The C index of OS predicted by the nomogram model was 0.928 (95% CI: 0.904–0.952). The bootstrap self-sampling method (B = 1000) was used for internal validation of the prediction model, and the calibration curve showed good agreement between the prediction and observation results of 1-year, 2-year, and 3-year OS. The ROC curves for 1-year, 2-year, and 3-year survival were plotted according to independent factors, and the AUC was 0.952 (95% CI: 0.925–0.979), 0.951 (95% CI: 0.916–0.985), and 0.939 (95% CI: 0.913–0.965), respectively. DCA shows that this model has good clinical application value.

**Conclusion:**

ALI can be used as a reliable indicator to evaluate the prognosis of patients with operable NSCLC, and through the construction of a nomogram model, it can facilitate better individualized treatment and prognosis assessment.

## Background

According to the results of the 2020 Global Cancer Epidemiology Survey, lung cancer has the second highest incidence among malignancies, but a high proportion of deaths, accounting for about 18% of all cancer-related deaths, is a major global public health problem [1]. Non-small-cell lung carcinoma (NSCLC) accounts for a high proportion of lung cancer tissue types, about 80–85%, and the 5-year survival rate is relatively low [2]. Biomarkers in the pre-treatment stage aid doctors in assessing patients’ prognosis and delivering personalized care during the perioperative period, thereby enhancing patient survival rates. Among them, inflammation plays a crucial role in the occurrence and development of cancer [3]. Previous studies have shown that inflammatory parameters can be used to assess the prognosis of cancer patients, such as serum C-reactive protein (CRP), neutrophil-to-lymphocyte ratio, platelet-to-lymphocyte rate (PLR), and neutrophil to lymphocyte ratio (NLR), which are two types of hematological indicators commonly used in clinical research and practice and have a good predictive value for evaluating the postoperative survival of NSCLC [4–8]. However, due to the limitations of a single indicator, it is not enough to reflect the systemic status of patients. The advanced lung cancer inflammation index (ALI) is determined by serum albumin (ALB), body mass index (BMI), and NLR, which constitute a composite index, and multiple studies have shown that ALI has good predictive value in assessing the survival prognosis of cancer patients [9–11]. However, the survival prognosis of cancer patients is affected by many factors, so it is necessary to find some potential factors early and deal with them in time. By constructing the nomogram prediction model, individual differences among patients can be observed more directly, which is helpful to provide a reference for individual treatment of potential risk factors.

## Material and method

### Object of study

The clinical data of 899 patients with operable and pathologically proven NSCLC admitted to our hospital from 2017 to 2021 was retrospectively analyzed. Inclusion criteria: (1) patients over the age of 18; (2) patients with pathologically proven NSCLC after thoracoscopic resection (stage I-III lung cancer according to TNM Edition 8 [12]); (3) no previous history of malignant tumors or the presence of a second primary cancer; (4) complete clinical data and follow-up are available. Exclusion criteria: (1) NSCLC patients who are unresectable or cannot tolerate surgical treatment; (2) patients with blood system diseases, immune system diseases, or blood abnormalities of unknown cause. (3) There are serious underlying diseases in the past (such as grade IV heart function, liver and kidney failure, stroke with serious sequelae, etc.), resulting in unclear outcome indicators; (4) Patients with missing data, incomplete return visit records (patients who failed to regularly return visit or refused to return visit), and patients who could not obtain their living status through telephone follow-up or refused to accept the investigation in this study.

### Data collection

Preoperative clinical data of patients meeting the inclusion criteria was collected by consulting the outpatient and inpatient electronic medical record systems of our hospital. Age, sex, smoking history, previous history of lung disease (bronchitis, bronchial asthma, bronchiectasis, etc.), Eastern Cooperative Oncology Group Performance Status Score (ECOG PS), pulmonary function indicators [forced expiratory volume in the first second (FEV_1_), forced vital capacity (FVC), and FEV_1_/FVC], tumor location, surgical protocol, tumor diameter, pathological type, TNM stage, Ki67 expression, and tumor invasion of blood vessels, lymphatic vessels, and nerves; the patient’s height and weight were measured, and the BMI (kg/m^2^) was calculated.

### Definition of ALI and its optimal truncation value

The patient had a normal body temperature 1 week before surgery and no symptoms of local or systemic infection. Venous blood was collected and sent to the clinical laboratory of our hospital for testing 1 week before surgery, and the results of neutrophil and lymphocyte and liver function albumin were recorded in the blood routine. Calculate ALI by multiplying BMI (kg/m^2^) by Alb (g/dl) and dividing by NLR[neutrophilic granulocyte (10^9^/L)/lymphocyte (10^9^/L)]. The receiver operating characteristic (ROC) curve determined the optimal truncation value for ALI. According to the optimal cut-off value of ALI, 899 patients were divided into a low ALI group and a high ALI group.

### Follow-up and definition of related concepts

The survival status of patients was assessed according to the hospital’s outpatient and inpatient record system and telephone. Overall survival (OS) was defined as the time from the date of pathologically diagnosed NSCLC to death or the end of the study (December 2022).

### Statistical method

SPSS 27.0 software and R4.2.1 software were used for data processing and analysis. The Kolmogorov-Smirnov method was used to test the normality of the measurement data. Those who met the normal distribution were represented by the positive and negative standard deviation of the mean, and a T-test was used for comparison between groups. Those who do not conform to the normal distribution are represented by *M (Q1, Q3)*, and the *U* test is applied for inter-group comparison. The count data were represented by the number of cases (%), and the comparison between groups was performed by a chi-square test or Fisher exact test. Survival analysis was presented by the Kaplan-Meier curve, and differences between groups were compared by the log-rank test.

The best cutoff values of tumor diameter and ALI were obtained by ROC curve, which were divided into two categorical variables. The predictors of OS were evaluated by Cox proportional risk model using single factor and stepwise regression multifactor analysis. Based on the results of multi-factor Cox proportional risk regression analysis, a nomogram model was established by using R survival package. The prediction performance of the nomogram model was verified by Bootstrap method (repeated sampling 1 000 times), and the concordance index (C-index) was used to represent the prediction performance of the nomogram model, and the calibration graph method was used to directly represent its prediction conformity. *P* < 0.05 was considered statistically significant.

## Results

### Baseline characteristics

This study conducted a comprehensive review of patients with NSCLC who underwent surgery in our hospital during the study period, and 899 patients were ultimately included in the analysis based on inclusion and exclusion criteria. Among the enrolled patients, the mean age of the patients was 62.0 ± 9.04 years old, ranging from 25 to 89 years old, including 413 males and 386 females. A total of 283 patients had a history of smoking, accounting for 31.5% of the sample. 220 patients (24.5%) had a history of pulmonary disease. In the index of lung function, 88 and 166 patients with FVC and FEV_1_ less than 80%, respectively, and 155 patients with FEV_1_ and FVC less than 70%. 179 cases had an ECOG PS score of 2 or above. The most common tumors were in the upper lobe of the left lung and the upper lobe of the right lung, accounting for 24.7% and 32.6% of the sample size, respectively. There were 597 cases of lobectomy, 184 cases of segmental resection, 112 cases of wedge resection, and 6 cases of total lung resection. The tumor diameter was < 2.25 cm in 645 cases and ≥ 2.25 cm in 254 cases. The pathological classification of NSCLC was adenocarcinoma in 793 cases and non-adenocarcinoma in 106 cases. There were 707, 87, and 105 patients in stages I, II, and III, respectively. The T stages were mainly T1–T2, accounting for 95.4% of the sample size. Most patients (82.6%) did not have lymph node metastasis. The expression of Ki67 was < 25% in 691 patients and ≥ 25% in 208 patients. There were 565, 552, and 24 cases of tumor vascular invasion, lymphatic vessel invasion, and nerve invasion, respectively. By the end of the study, 97 patients had died.

The patients were divided into two groups according to whether there was death or not, and the ROC curve was described. The optimal cutoff value of ALI, a complex indicator of inflammation and nutrition, was 70.06. The best truncation value divides ALI into two categorical variables. 592 patients (65.9%) had ALI levels below the cutoff value, and 307 patients (34.1%) had ALI levels above the cutoff value. Among them, male patients (*P* < 0.001), previous smoking history (*P* < 0.001), previous history of lung disease (*P* = 0.005), FVC < 80% (*P* = 0.033), FEV_1_ < 80% (*P* = 0.005), FEV_1_ / FVC < 70% (*P* = 0.002), ECOG PS score ≥ 2 points (*P* = 0.049), lobectomy (*P* = 0.001), tumor diameter ≥ 2.25 cm (*P* < 0.001), non-adenocarcinoma (*P* < 0.001), pathological stage I-II (*P* < 0.001), T2-3 (*P* = 0.006), N0 and N2 (*P* < 0.001) and Ki67 expression ≥ 25% (*P* < 0.001) were higher in the low ALI group. No statistical difference was found in other demographic and clinical characteristics, as shown in Table 1.

**Table 1:** Patient characteristics [n (%)]

Baseline	Low ALI (< 70.06) (*n* = 592)	High ALI (≥ 70.06) (*n* = 307)	*P*
**Age (years)**			
< 65	359 (60.6)	202 (65.8)	0.146
≥ 65	233 (39.4)	105 (34.2)	
**Gender**			
Male	319 (53.9)	94 (30.6)	< 0.001
Female	273 (46.1)	213 (49.3)	
**Smoking status**			
Never smoking	367 (62.0)	249 (81.1)	< 0.001
Current or former smoker	225 (38.0)	58 (18.9)	
**Respiratory diseases**			
Without	430 (72.6)	249 (81.1)	0.005
With	162 (27.4)	58 (18.9)	
**FVC (%)**			
<80	67 (11.3)	21 (6.8)	0.033
≥ 80	525 (88.7)	286 (93.2)	
**FEV**_**1**_ **(%)**			
<80	125 (21.1)	41 (13.4)	0.005
≥ 80	467 (78.9)	266 (86.6)	
**FEV**_**1**_ **/ FVC (%)**			
<70	119 (20.1)	36 (11.7)	0.002
≥ 70	473 (79.9)	271 (88.3)	
**ECOG PS score**			
0–1	469 (79.2)	260 (84.7)	0.049
≥ 2	123 (20.8)	47 (15.3)	
**Tumor location**			0.875
superior lobe of left lung	147 (24.8)	75 (24.4)	
inferior lobe of left lung	92 (15.5)	56 (18.2)	
superior lobe of right lung	197 (33.3)	96 (31.3)	
middle lobe of right lung	53 (9.0)	26 (8.5)	
inferior lobe of right lung	103 (17.4)	54 (17.6)	
**Operative programme**			0.001
pulmonary lobectomy	419 (70.8)	178 (58.0)	
segmentectomy	102 (17.2)	82 (26.7)	
wedge resection	66 (11.1)	46 (15.0)	
total pneumonectomy	5 (0.8)	1 (0.3)	
**Maximum tumor diameter**			
< 2.25	398 (67.2)	247 (80.5)	< 0.001
≥ 2.25	194 (32.8)	60 (19.5)	
**Pathologic types**			
Adenocarcinoma	499 (84.3)	294 (95.8)	< 0.001
Non-Adenocarcinoma	93 (15.7)	13 (4.2)	
**Pathological stage**			< 0.001
stage I	436 (73.6)	271 (88.3)	
stage II	70 (11.8)	17 (5.5)	
stage III	86 (14.5)	19 (6.2)	
**T-staging**			0.006
T_1_	341 (57.6)	203 (66.1)	
T_2_	215 (36.3)	99 (32.2)	
T_3_	25 (4.2)	4 (1.3)	
T_4_	11 (1.9)	1 (0.3)	
**N-staging**			< 0.001
N_0_	467 (78.9)	0 (0.0)	
N_1_	47 (7.9)	276 (89.9)	
N_2_	73 (12.3)	12 (3.9)	
N_3_	5 (0.8)	19 (6.2)	
**Ki67**			
< 25%	422 (71.3)	269 (87.6)	< 0.001
≥ 25%	170 (28.7)	38 (12.4)	
**Vascular invasion**			
Without	216 (36.5)	118 (38.4)	0.610
With	376 (63.5)	189 (61.6)	
**Lymphatic vessel invasion**			
Without	228 (38.5)	119 (38.8)	0.943
With	364 (61.5)	188 (61.2)	
**Nerve invasion**			
Without	575 (97.1)	300 (97.7)	0.669
With	17 (2.9)	7 (2.3)	

Note: ALI: the advanced lung cancer inflammation index; FVC: Forced vital capacity; FEV1: Forced expiratory volume in the first second; ECOG PS: Eastern Cooperative Oncology Group Performance Status Score

**Fig. 1:**
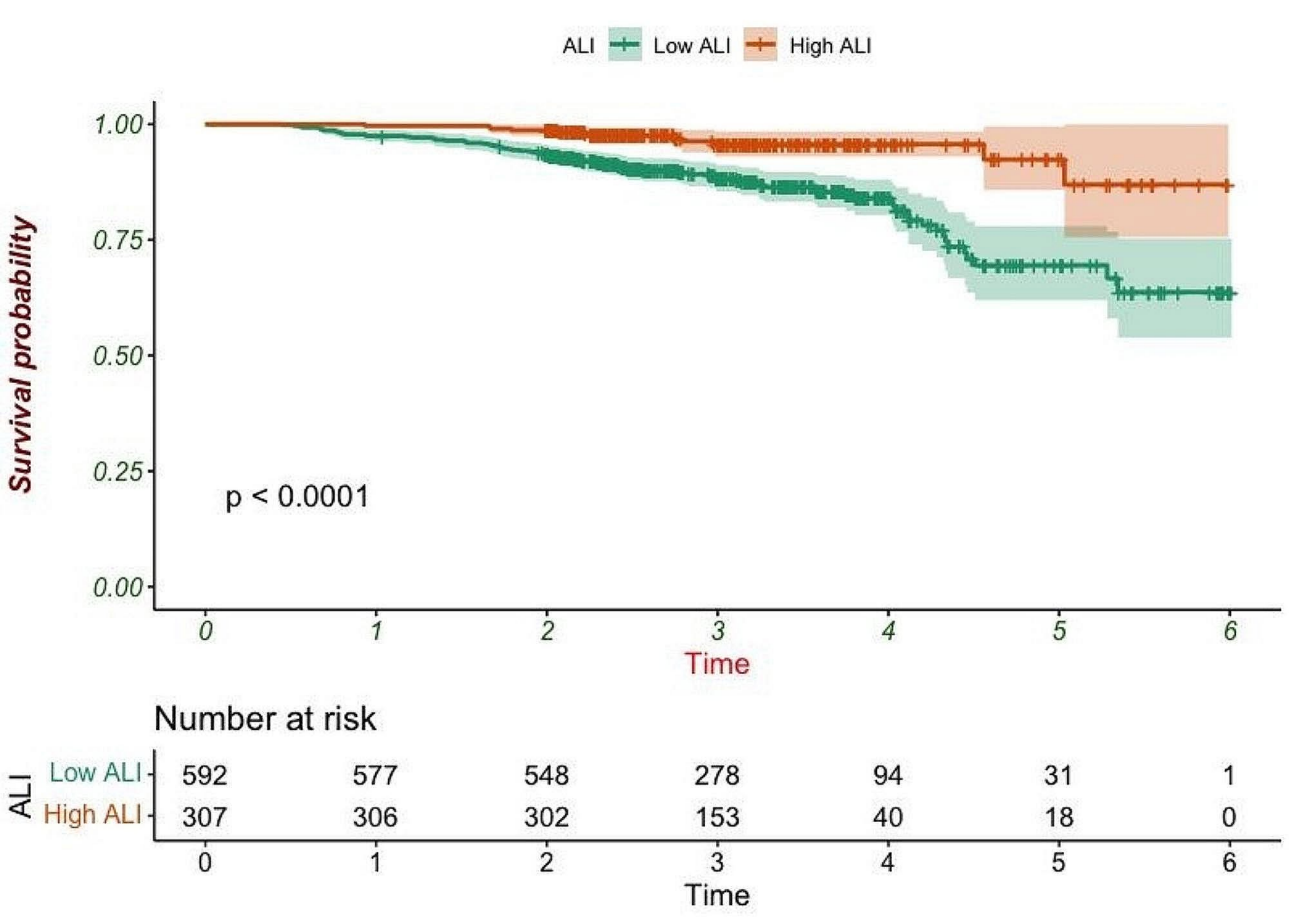
Kaplan-Meier survival curves of patients with different ALI levels

Survival analysis was performed based on ALI levels, and the Kaplan-Meier survival curve showed a significant difference in survival between the two groups (Fig. 1). The survival rate of the low-ALI group was significantly lower than that of the high-ALI group (*P* < 0.0001).

### Univariate and multivariate regression analysis of clinical features and the relationship between ALI and OS

Overall survival was taken as a time variable, death was taken as a state variable (yes, “1,” no, “0”), and factors such as patient characteristics and ALI were included as independent variables in the Cox proportional risk model for univariate analysis. Gender, smoking status, tumor location, surgical protocol, tumor diameter, pathological type, pathological stage, T stage, N stage, Ki67 expression, nerve invasion, and ALI were correlated with the prognosis of NSCLC patients (*P* < 0.05). The multivariate Cox regression model incorporated the above statistically significant factors, revealing that tumor location in the lower lobe of the right lung (HR: 2.834, 95%CI 1.476–5.443, *P* = 0.002), stage II (HR: 68.090, 95%CI 12.583-368.456, *P* < 0.001), stage III NSCLC lung cancer (HR: 27.804, 95%CI 8.235–93.882, *P* < 0.001), and neuroaggression (HR: 2.240, 95%CI 1.019–4.924, *P* = 0.045) were identified as independent risk factors for overall survival of NSCLC patients. The level of ALI (above the cutoff value) (HR: 0.443, 95%CI: 0.231–0.850, *P* = 0.014) was the prognostic factor for improved overall survival in NSCLC patients. As shown in Table 2.

**Table 2:** Univariate and multivariate analysis of overall survival in NSCLC patients

Category	Overall Survival							
	*n* (%)	Univariate Analysis				Multivariate Analysis		
		HR	95% CI	*P* - value		HR	95% CI	*P* - value
**Age (years)**								
< 65	561 (62.4)		1.000					
≥ 65	338 (37.6)	1.358	0.908∼2.031	0.136				
**Gender**								
Male	413 (45.9)		1.000				1.000	
Female	486 (54.1)	0.593	0.395∼0.891	0.012		0.636	0.350∼1.156	0.138
**Smoking status**								
Never smoking	616 (68.5)		1.000				1.000	
Current or former smoker	283 (31.5)	1.885	1.264∼2.812	0.002		0.763	0.446∼1.307	0.325
**Respiratory diseases**								
Without	679 (75.5)		1.000					
With	220 (24.5)	1.401	0.910∼2.157	0.126				
**FVC (%)**								
<80	88 (9.8)		1.000					
≥ 80	811 (90.2)	0.618	0.361∼1.058	0.079				
**FEV**_**1**_ **(%)**								
<80	166 (18.5)		1.000					
≥ 80	733 (81.5)	0.660	0.421∼1.036	0.071				
**FEV**_**1**_ **/ FVC (%)**								
<70	155 (17.2)		1.000					
≥ 70	744 (82.8)	0.790	0.478∼1.307	0.359				
**ECOG PS score**								
0–1	729 (81.1)		1.000					
≥ 2	170 (18.9)	1.195	0.730∼1.955	0.479				
**Tumor location**								
superior lobe of left lung	222 (24.7)		1.000				1.000	
inferior lobe of left lung	148 (16.5)	1.440	0.755∼2.747	0.269		1.315	0.664∼2.603	0.433
superior lobe of right lung	293 (32.6)	1.835	0.866∼3.889	0.113		1.148	0.499∼2.642	0.746
middle lobe of right lung	79 (8.8)	0.925	0.504∼1.699	0.802		1.135	0.579∼2.225	0.713
inferior lobe of right lung	157 (17.5)	2.034	1.103∼3.753	0.023		2.834	1.476∼5.443	0.002
**Operative programme**								
pulmonary lobectomy	597 (66.4)		1.000				1.000	
segmentectomy	184 (20.5)	1.935	0.476∼7.867	0.356		0.463	0.103∼2.072	0.314
wedge resection	112 (12.5)	0.206	0.065∼0.650	0.007		1.297	0.375∼4.488	0.681
total pneumonectomy	6 (0.7)	0.148	0.047∼0.471	0.001		2.749	0.746∼10.122	0.128
**Maximum tumor diameter**								
< 2.25	645 (71.7)		1.000				1.000	
≥ 2.25	254 (28.3)	4.524	2.989∼6.849	< 0.001		0.839	0.513∼1.373	0.485
**Pathologic types**								
Adenocarcinoma	793 (88.2)		1.000				1.000	
Non-Adenocarcinoma	106 (11.8)	2.362	1.478∼3.773	< 0.001		1.415	0.806∼2.487	0.227
**Pathological stage**								
stage I	707 (78.6)		1.000				1.000	
stage II	87 (9.7)	106.771	46.118∼247.190	< 0.001		68.090	12.583∼368.456	< 0.001
stage III	105 (11.7)	31.486	13.033∼76.066	< 0.001		27.804	8.235∼93.882	< 0.001
**T-staging**								
T_1_	544 (60.5)		1.000				1.000	
T_2_	314 (34.9)	19.450	8.270∼45.745	< 0.001		1.221	0.417∼3.570	0.716
T_3_	29 (3.2)	8.058	3.808∼17.052	< 0.001		1.228	0.483∼3.122	0.667
T_4_	12 (1.3)	3.693	2.250∼6.063	< 0.001		1.429	0.808∼2.528	0.219
**N-staging**								
N_0_	743 (82.6)		1.000				1.000	
N_1_	59 (6.6)	75.332	25.123∼225.879	< 0.001		3.373	0.603∼18.869	0.166
N_2_	92 (10.2)	40.410	23.350∼69.936	< 0.001		2.077	0.543∼7.938	0.285
N_3_	5 (0.6)	13.078	6.907∼24.762	< 0.001		1.079	0.457∼2.551	0.862
**Ki67**								
< 25%	691 (76.9)		1.000				1.000	
≥ 25%	208 (23.1)	3.104	2.082∼4.628	< 0.001		1.080	0.676∼1.725	0.749
**Vascular invasion**								
Without	334 (37.2)		1.000					
With	565 (62.8)	1.293	0.849∼1.970	0.231				
**Lymphatic vessel invasion**								
Without	347 (38.6)		1.000					
With	552 (61.4)	1.283	0.848∼1.940	0.239				
**Nerve invasion**								
Without	875 (97.3)		1.000				1.000	
With	24 (2.7)	3.559	1.725∼7.342	0.001		2.240	1.019∼4.924	0.045
**ALI**								
< 70.06	592 (65.9)		1.000				1.000	
≥ 70.06	307 (34.1)	0.271	0.148∼0.496	< 0.001		0.443	0.231∼0.850	0.014

Note: ALI: the advanced lung cancer inflammation index; FVC: Forced vital capacity; FEV1: Forced expiratory volume in the first second; ECOG PS: Eastern Cooperative Oncology Group Performance Status Score

### Establishment and verification of nomogram model

**Fig. 2:**
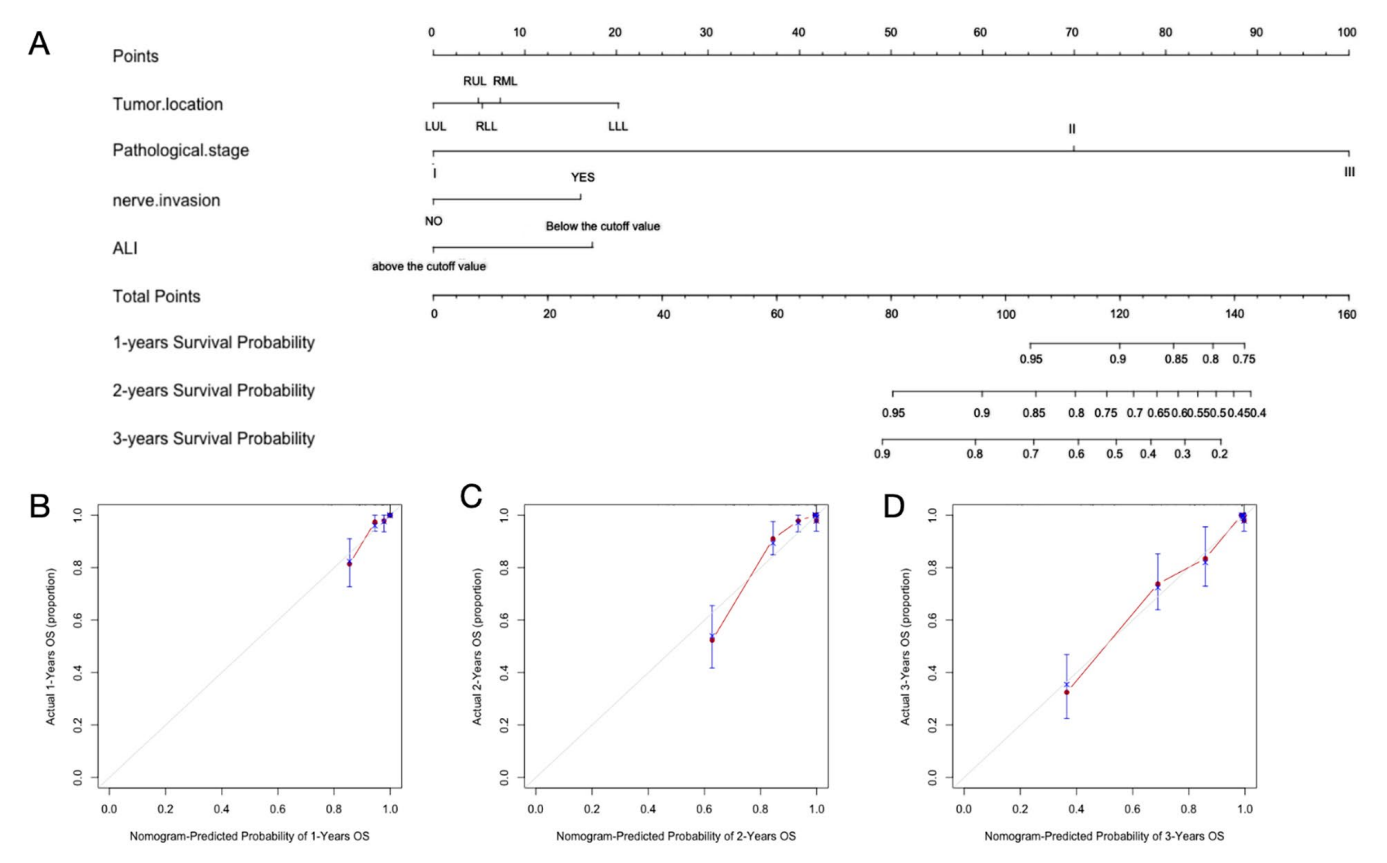
Nomogram and calibration curve for predicting overall survival of NSCLC patients. (**A**) Nomogram model; calibration curves for 1-year (**B**), 2-year (**C**), and 3-year (**D**) survival rates

In order to visually represent the prediction results of the model, R software was used to draw a nomogram model based on statistically significant variables (tumor location, pathological stage, nerve invasion, and ALI level) in Cox regression analysis. The model was used to evaluate individualized prognostic predictions in patients with surgically treated NSCLC. As shown in Fig. 2A. The score of the first row corresponding to the vertical of each index is added to obtain the total score, which can intuitively determine the estimated survival probability of patients in 1 year, 2 years, and 3 years. The higher the score, the worse the predicted prognosis.

The prediction performance of the nomogram model was evaluated by C-index and calibration curve, and the results showed that the model predicted OS with a C-index of 0.928 (95% CI: 0.904–0.952). The Bootstrap self-sampling method (B = 1000) was used to internally verify the prediction model. The predicted survival rate was taken as the horizontal coordinate and the actual survival rate as the vertical coordinate. The calibration curve showed that there was good consistency between the predicted survival rate and the actual observation probability of NSCLC patients after 1, 2, and 3 years. It shows that the model fits well. As shown in Fig. 2B and D.

**Fig. 3:**
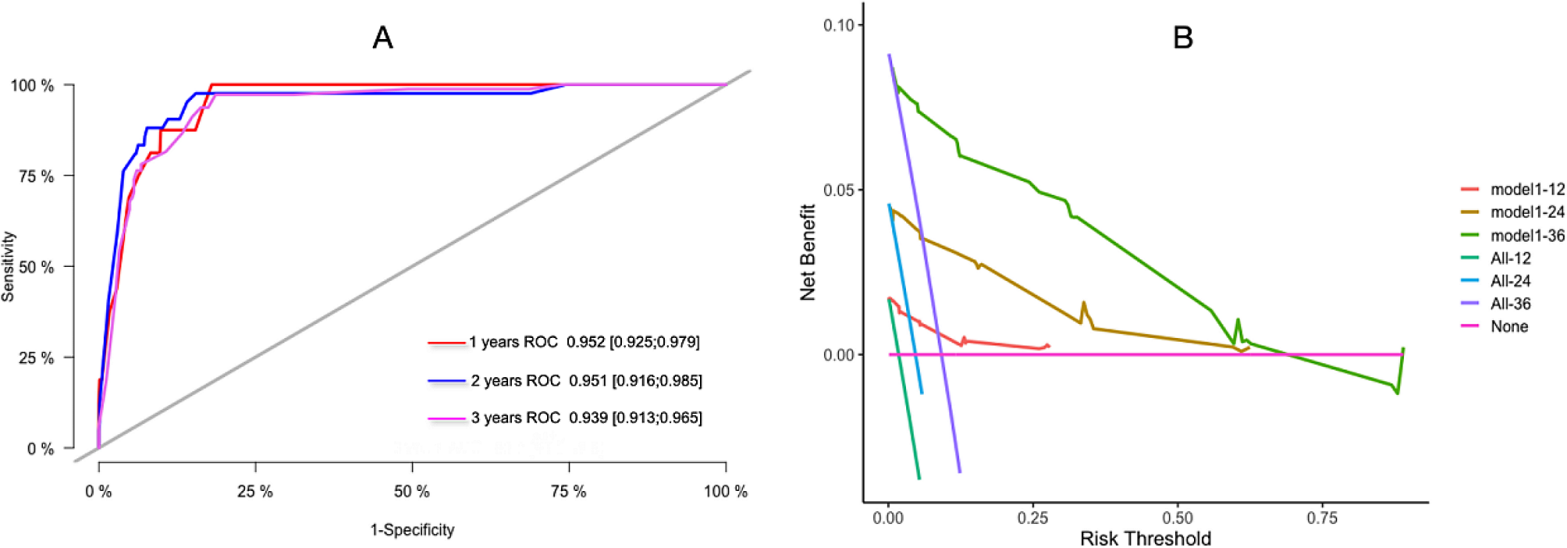
ROC curve and DCA curve of prediction model. (**A**) ROC curves for 1-, 2-, and 3-year survival predictions; (**B**) 1-year, 2-year, and 3-year clinical value DCA curves

ROC curves for 1, 2, and 3-year survival rates were plotted according to independent factors to evaluate the accuracy of the model. The results showed that the area under curve (AUC) of the model was 0.952 (95% CI: 0.925–0.979) and 0.951 (95% CI: 0.916–0.985), respectively, and 0.939 (95% CI: 0.913–0.965). The model showed good differentiation (Fig. 3A). Decision curve analysis (DCA) was used to evaluate the application value of the model, and the results showed that when the threshold probability was greater than 0.05, the threshold probability was positively correlated with the net benefit level of the model, as shown in Fig. 3B.

## Discussion

The prognosis varies significantly among different phases of TNM. A number of studies have confirmed that the 3-year survival rate of stage I lung cancer can reach more than 90%, while the 3-year survival rate of stage II lung cancer can reach about 70%. The survival rate for stage III lung cancer patients is usually less than 50%. In addition, disease recurrence and metastasis occurred mainly within the first two years after surgical intervention. This has led to a significant decline in survival for stage III patients [13–15]. Therefore, patients should be closely followed up after surgery, with timely detection of tumor recurrence and metastasis and timely diagnosis and treatment intervention to improve their survival prognosis and quality of life.

The relationship between tumor location and prognosis was related to the distribution of lymph node metastasis. Yang et al. analyzed the association between mediastinal lymph node metastasis distribution and survival in patients with operable NSCLC (≤ 3 cm). The results were as follows: upper right lobe, 4R station (17.7%); right middle lobe, 7th station lymph node (14.9%); right inferior lobe, 7th station lymph node (19.8%); left upper lobe, 7th station lymph node (16.6%); left upper lobe, 5th station lymph node (18.2%) [16]. The Guo et al.‘s study explored the association between the primary site and the mediastinal lymph node station in patients undergoing radical resection of N2 lymph node metastases and showed that the highest rate (100%) was found in lymph node station 2/4, occurring in the right upper lobe. The proportion of lymph nodes at the 7th station in the right middle lobe and lower lobe was higher, accounting for 80% and 88.9%, respectively. The left upper lobe mainly occurred in the 5th station lymph node (84.4%) [17]. Lin et al. evaluated the prognosis of the inferior lobe (basal and superior segments) in patients with operable lung adenocarcinoma. The results showed that N2 lymph node metastasis was more likely to occur in the basal segment than in the upper segment of lung adenocarcinoma excised in the lower lobe [18]. In Liu et al.‘s study, inferior lobe origin was strongly associated with lower overall survival compared to non-right inferior lobe tumors [19].

Since Virchow proposed the hypothesis that cancer occurs at the site of chronic inflammation, extensive research has been conducted on the correlation between inflammation and cancer. The tumor microenvironment plays a crucial role in the occurrence and development of tumors by promoting cell proliferation and migration under the regulation of inflammatory cells [20]. Prior retrospective research has demonstrated a strong correlation between NLR and survival rates in patients with operable NSCLC [8, 21]. The meta-analysis verified that an elevated NLR is an indicator of unfavorable outcomes in patients with lung cancer [5, 22]. The possibility that NLR can effectively assess survival in patients with NSCLC is explained by the fact that neutrophils play an important role in all aspects of cancer progression, including tumor initiation, growth, proliferation, and progression. Neutrophils are involved in tumor-related properties by promoting angiogenesis, motility, migration, and invasion, as well as regulating other immune cells [23]. In addition, some neutrophils have the ability to induce epithelial mesenchymal transformation through the TGF-β/Smad signaling pathway, which is also considered to be a key factor in tumor occurrence and development [24]. This relationship places neutrophils in a central role in tumor inflammation, playing an important role in tumor growth and progression through their direct effects on tumor cells or through their indirect effects on the tumor microenvironment. Studies have shown that high levels of neutrophils are linked to decreased survival in NSCLC [25, 26]. Lymphocytes can hinder tumor cell growth and movement by releasing cytokines, which significantly contribute to tumor defense and immunological monitoring [27]. According to research, a decrease in the percentage of lymphocytes in peripheral blood may result in an elevation in the NLR, which is strongly associated with disease progression [28].

Due to the characteristics of rapid metabolism and proliferation of tumor cells, nutritional status indicators such as BMI and albumin are also important clinical prognostic parameters for evaluating lung cancer treatment. Malnutrition has been observed in previous studies to be associated with poorer overall survival, time to tumor progression, and quality of life in lung cancer patients [29–33]. It is important to note that anti-cancer treatments, including surgery, may exacerbate the severity of malnutrition. In addition, malnutrition is associated with increased susceptibility to perioperative morbidity and death. Incorporating nutritional assessment into pre-treatment regimens for cancer patients is critical, as research has shown that providing nutritional support can effectively mitigate the adverse effects of malnutrition on perioperative outcomes [34, 35].

Compared with NLR, PLR, and other single indicators, ALI is a complex indicator composed of inflammation and nutrition indicators, which can more comprehensively assess the level of inflammation, immune function, and nutritional status of patients. This approach has been shown to be beneficial for a more effective assessment of patient outcomes. Song et al. [36] and colleagues assessed the prognosis value of 16 inflammatory and nutritional markers for OS in patients with lung cancer. They discovered that the prognostic ability of ALI was superior to other inflammation and nutrition indicators. Mandaliya et al. [37] assessed the prognostic significance of NLR, ALI, PLR, and lymphocyte-to-monocyte ratio (LMR) in predicting OS in patients with advanced NSCLC. The study revealed a strong correlation between NLR and ALI with OS. Mountzios et al. [38] also verified that ALI serves as a robust indicator for forecasting survival outcomes in patients with advanced NSCLC who are undergoing treatment with immune checkpoint drugs. Additionally, this study demonstrates that ALI possesses a robust capacity to forecast outcomes in patients with NSCLC who are undergoing surgical procedures. Therefore, Assessment of ALI levels before treatment, stratification of patients who may have low levels of ALI, and adjustment of individualized treatment regimens (for example, low BMI and ALB indicate poor nutrition and can be given appropriate nutritional support) can help to prolong patient survival and improve quality of life. Second, ALI offers several advantages, including simple measurement, routine availability, and a high degree of standardization. Therefore, we suggest that ALI may serve as a reliable and cost-effective prognostic indicator of postoperative survival in patients with NSCLC.

Lymphatic vascular invasion (LVI) has long been associated with poorer survival and highly aggressive tumors [39]. In the study of Dicken BJ et al., LVI is considered to be an effective factor that can independently predict survival and is correlated with T stage [40]. LVI has been observed as a significant predictor of OS in retrospective studies of gastric cancer and NSCLC [41–43]. However, no correlation was found between LVI and OS in this study, which may be because LVI mostly appeared in middle- and advanced-stage patients, while this study mainly focused on patients with stages T1–T2, and the risk of LVI was relatively low. In addition, perineural invasion (PNI) is also a strong predictor of postoperative NSCLC. In the study of Demir A et al., PNI was observed to be a decisive factor for poor prognosis. According to their report, the presence of PNI was found to have a significant negative impact on 3-year and 5-year survival (3-year survival decreased from 54 to 32% and 5-year survival decreased from 15–0%) [44]. Kilicgun A et al.‘s report observed that patients with stage IA with perineural infiltration had a worse survival rate than patients without PNI but with stage IIIA disease [45]. Another retrospective study also confirmed that the appearance of PNI significantly shortened the patient’s prognosis. The 3-year survival rates of stage I NSCLC patients with and without PNI were 23.3% and 63.2%, respectively [46]. Our results showed that the occurrence of PNI could increase the risk of death by 2.24 times, and PNI still had good predictive value after excluding multiple confounding factors.

The traditional TNM staging system ignores the biological differences between tumors and normal tissues. Compared with the TNM stage prognostic evaluation system, the nomogram can synthesize various prognostic indexes and has a better prognostic evaluation value [47]. Each independent variable receives a score based on the specific patient situation, and the nomogram’s high accuracy and practicability allow for the calculation of the probability of patients’ occurrence of outcome events based on the relationship between the total score and the probability of outcome occurrence. Secondly, compared with simple models and scoring systems that contain only a few variables, the nomogram model can more comprehensively analyze the risk factors and interaction effects related to survival prognosis, which is of great significance to provide guidance for the clinical management of patients. Based on the above factors, the nomogram has an important clinical reference value in predicting the prognosis of NSCLC patients.

In this study, the prognosis of patients with operable NSCLC was evaluated by constructing a nomogram model. The model was evaluated by the C-index and correction curve, and the results showed that the model had high prediction accuracy and clinical practicability and could provide an important clinical reference value for the prognosis assessment of NSCLC. However, there are still some limitations: First of all, this is a retrospective analysis with a single-center design and limited sample size, which inevitably leads to selection bias in the selection of study subjects and clinical data collection. Secondly, there is no clear consensus on the optimal cut-off value of ALI. The effect of dynamic changes in ALI values on the long-term prognosis remains to be evaluated. Moreover, any underlying factors that affect changes in blood indicators (such as previous nutritional support in other hospitals) can lead to fluctuations in ALI results. Third, this study lacks external data to verify the model. The essence of the above reasons lies in the fact that in the retrospective study, the pre-treatment status of patients (including whether they had received nutritional support in other hospitals and whether there were infection factors that had been treated in other hospitals before treatment) can only be judged by the hospitalization log recorded in the electronic case system, which inevitably causes selection bias. Therefore, prospective studies need to strictly include and exclude patients to avoid deviations from ALI results. Secondly, studies conducted by individual institutions are more limited, and the results may only be representative of the region, with regional differences (such as the relatively poor nutritional status of patients in some economically disadvantaged areas). Therefore, we need multi-center studies to control the limitation of regional populations. Based on the above reasons, it is necessary to conduct multi-center and prospective studies in the future to improve the model so that the built columns can predict the prognosis of patients more accurately and gain benefits for both doctors and patients.

## Conclusion

Low ALI can identify patients with a poor prognosis. Based on the results of multi-factor analysis, the nomogram model can effectively predict the survival of patients and provide a reference for individual treatment. ALI can be a valuable marker of prognosis in patients with operable non-small cell lung cancer.

## Data Availability

No datasets were generated or analysed during the current study.
